# Photoluminescence Properties of Polymorphic Modifications of Low Molecular Weight Poly(3-hexylthiophene)

**DOI:** 10.1186/s11671-017-2134-5

**Published:** 2017-05-23

**Authors:** Takashi Kobayashi, Keita Kinoshita, Akitsugu Niwa, Takashi Nagase, Hiroyoshi Naito

**Affiliations:** 1 Department of Physics and Electronics, Osaka Prefecture University, 1-1 Gakuencho, Nakaku, Sakai, Osaka 599-8531 Japan; 2 The Research Institute of Molecular Electronic Devices, Osaka Prefecture University, 1-1 Gakuencho, Nakaku, Sakai, Osaka 599-8531 Japan

**Keywords:** P3HT, Molecular weight, Polymorph, Photoluminescence, Intermolecular interactions

## Abstract

The structural and photoluminescence (PL) properties of thin films of poly(3-hexylthophene) (P3HT) with molecular weights (MWs) of 3000 and 13,300 have been investigated. Although high MW P3HT always self-organizes into one packing structure (form I), low MW P3HT forms two different packing structures (forms I and II) depending on the fabrication conditions. In this work, several fabrication techniques have been examined to obtain form II samples with little inclusion of a form I component. It is found that drop-cast thin films of low MW P3HT (form II) exhibit a PL spectrum that is different from that of form I and does not contain the form I component. The PL spectrum can thus be attributed to form II. The differences in PL properties between forms I and II can be understood in terms of weakened interchain interactions due to the longer interchain distance in form II.

## Backgrounds

Poly(3-alkylthiophene)s (P3ATs), which are representative π-conjugated polymers, are known to occur in more than two different crystalline structures depending on the processing conditions [1–15]. High molecular weight (MW) P3ATs usually form a lamellae π-stacking structure (form I) where fully planar backbones stack face to face with a stacking distance of 3.8 Å [3, 6, 9, 11, 16]. Because of such a short distance, charge states in form I are delocalized over several backbones [16–18]. On the other hand, solid-state samples of low MW P3ATs often exhibit also a different packing structure (form II) [3, 6, 9], in which the distance between the nearest-neighbor backbones increases up to 4.4 Å due to tilted and interdigitated alkyl chains [2, 3, 12–14]. Such differences in the crystalline structure are naturally expected to alter the optoelectronic properties. However, the difference in the optical properties, in particular the photoluminescence (PL), between the form I and II modifications has not yet been revealed. This may be due to the difficulty in preparation of form II samples whose quality is high enough for PL studies; actual form II samples usually contain significant fractions of form I modifications as well as amorphous backbones.

Recently, Lu et al. have found that formation of form II of poly(3-butylthiophene) (P3BT) is promoted by slowly evaporating a disulfide solvent or exposing the sample to a disulfide vapor (a vapor treatment) [7, 8]. Using the fact that form II of P3ATs is converted into form I by thermal annealing [2, 3, 9, 15], Lu et al. have demonstrated the reversible transformation between form I and II modifications of P3BT. Interestingly, such behavior is very similar to the phase transition of poly(9,9-dioctylfluorene) (F8) [19–24]; the crystalline phase of F8 is prepared by thermal annealing whereas β-phase F8 appears after exposure of the samples to a vapor of a good solvent. The reversible transformation between crystalline and β phases has also been confirmed [23, 24]. In the case of F8, high-quality β-phase thin films are prepared by dropping a dilute solution onto a substrate and waiting for a few hours to evaporate the solvent (drop-casting) [22]. Since there are many similarities between P3BT and F8 in spite of their entirely different backbone structures, it may be expected on the analogy with F8 that better quality form II thin films of P3ATs can be prepared by drop-casting.

In this work, we have fabricated thin films of poly(3-hexylthiophene) (P3HT) with several MWs by using several techniques, including drop-casting, and investigated their structural and optical properties. We have chosen P3HT in this work because, compared to P3BT, more data for P3HT is available in the literature. Among form II modifications obtained in this work using P3HT with MW = 3000, the ones prepared by drop-casting are the most suitable for PL measurements as we expected; the PL component of the other form is largely suppressed in the observed PL spectrum. We also discuss the mechanisms of the formation of the form II modifications and of their PL spectral differences.

## Methods

Regioregular P3HTs with different molecular weights were purchased and used as received. Their average MW and polydispersity index (PDI) were determined by gel permeation chromatography referred to polystyrene standards. Among those P3HTs, here, we report the results of P3HTs with MW = 3000 (PDI = 1.3) and MW = 13,300 (PDI = 1.3), and we hereafter refer them to low and high MW P3HTs, respectively. Note that a single P3HT chain with MW = 3000 consists of nearly 20 thiophene rings.

Thin films were fabricated by spin-coating or drop-casting from chloroform solutions onto quartz substrates, which were simply ultrasonically cleaned in several organic solvents. The P3HT concentrations of the solutions were controlled so that the resultant film thickness is in a range from 80 to 120 nm. To remove residual solvents, all the thin films were dried in a vacuum for 30 min. For some of the thin films, thermal annealing at 155 °C for 30 min was carried out in a vacuum. Vapor treatment was performed by exposing some of the thin films to a saturated atmosphere of chloroform vapor for 15 h. For XRD studies, in addition to those thin films, we prepared a precipitate of low MW P3HT that was obtained by adding a large amount of poor solvent, i.e., methanol, into the chloroform solution and this was then dried on a Si substrate.

The absorption spectra of the thin films were measured at 6 K with an optical multichannel analyzer equipped with a calibrated CCD detector and a Xenon lamp. The PL spectra were measured at 6 K with the optical multichannel analyzer and a green diode laser (532 nm). For the measurements of the excitation spectra, we used a double monochromator and a high-power Xenon lamp instead of the green diode laser. During the absorption and PL measurements, the samples were maintained in a vacuum with a closed cycle He cryostat. The out-of-plane XRD measurements were carried out at ambient atmosphere with a diffractometer using Cu Kα radiation.

## Results and Discussion

**Fig. 1:**
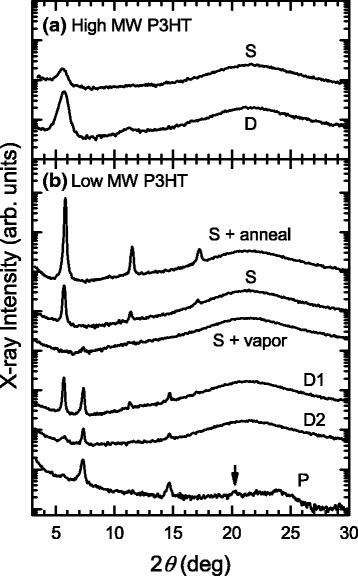
Out-of-plane XRD patterns of **a** high and **b** low MW P3HTs. The *arrow* indicates the small reflection around 20.2°. *S*, *D*, and *P* mean the samples prepared by spin-coating, drop-casting, and precipitation, respectively. For more details of fabrication methods see the text. The extremely broad peak centered on 22° is a halo of quartz substrates. The patterns are vertically offset for clarity

Figure 1a shows the out-of-plane XRD patterns of thin films of high MW P3HT. The observed patterns are typical of thin films of form I, in which the first and sometimes higher-order diffractions due to the separation between π stacks are observed [1–4, 6, 9]. The lack of a diffraction around 22° corresponding to the stacking distance of 3.8 Å indicates that, in those thin films, the stacking direction is parallel to the substrate. In the case of high MW P3HT, the packing structure is independent of the fabrication method used.

As shown in Fig. 1b, spin-coated and annealed thin films of low MW P3HT also exhibit XRD patterns characteristic of form I. On the other hand, an additional series of diffractions at 7.35° and 14.7° emerges in the XRD patterns of drop-cast thin films of low MW P3HT. Those diffraction angles are in good agreement with the values reported for the form II modification of P3HT [6, 9]. The mixing ratio of forms I and II is sensitive to the processing conditions (see D1 and D2 in Fig. 1b). As the evaporation of the solvent is slowed down, the relative intensity of the diffraction features corresponding to form II increases. Form II samples of low MW P3HT can also be prepared by exposing form I samples, e.g., spin-coated thin films, to a chloroform vapor. This result indicates that the reversible transformation between the form I and II modifications is possible with low MW P3HT. Note that the vapor-treated samples of low MW P3HT had rough surfaces. The resultant low coverage of the polymer on the quartz substrate accounts for the lower diffraction intensity (see S + vapor in Fig. 1b). In order to further confirm the formation of form II, we prepared a precipitate of low MW P3HT, in which the stacking direction is expected to be randomly oriented. Such a sample indeed shows diffraction at 20.2°, which represents the separated stacking distance of 4.4 Å in form II [2, 3, 6, 9].

Before showing their optical properties, we discuss a possible mechanism of the polymorphic behavior of P3ATs. The existence of the polymorphic modifications indicates that the energetic stabilities of the two packing structures are very similar. Since the polythiophene backbones and alkyl chains adopt fully planar and all-trans conformations, respectively, at room temperature [25, 26], the stability of a packing structure is determined by nonbonded attractions between polymer backbones and between alkyl chains [27–29]. The ones between a backbone and an alkyl chain are minor so that they are usually ignored [28, 29]. From the observations, it seems to be reasonable to consider that in form I and II modifications, a different type of those attractions largely contribute to the stabilization. Let us consider the formation of form II during a drop-casting process. Chloroform is a good solvent for alkyl chains but polythiophene backbones are intrinsically insoluble in organic solvents. Therefore, before the chloroform evaporates completely, there exists a time period in which the backbones attempt to form a packing structure while alkyl chains are still dissolved. If such a time period is long enough, as in a drop-casting process, the polymer chains self-organize into a packing structure where attractions between the backbones are preferred to those between alkyl chains (form II). On the other hand, thermal annealing influences equally the backbones and alkyl chains, and thus results in the thermodynamically most favored packing structure (form I) [13, 30].

This scenario consistently explains several experimental observations. For example, spin-coated thin films always become form I because the time period in which only alkyl chains are dissolved is too short to allow form II to appear. As far as we examined, the formation of form II was not recognized in samples of P3HT with MWs larger than or equal to 5200. Also in the literature, the form II modifications were obtained only for low MW P3HT [6, 9]. The number of alkyl chains attached to the single backbone is approximately proportional to its MW, and consequently, the stabilization due to the crystallization of alkyl chains increases in step with the MW. On the other hand, nonbonded attractions between the polymer backbones are not proportional to the chain length. The van der Waals force is considered to increase with a chain length but this proportionality is valid only for a short-chain regime. In a longer chain regime, the van der Waals force gradually becomes less MW dependent as the chain length increases and finally approaches the particular value of an infinite chain. This can be confirmed from the relationship between a melting point of polyethylene and its MW [31]. Therefore, although attractions between the polymer backbones and between alkyl chains compete with each other in low MW P3HT, high MW P3HT always form a packing structure where attractions between alkyl chains are preferred (form I). What would be expected for P3ATs with shorter alkyl chains, such as P3BT? Nonbonded attractions between butyl chains are weaker than those between hexyl chains. Thus, the two types of attractions would be balanced over a range of even longer MW. This explains why Lu et al. have obtained form II samples of P3BT with relatively large MW [8].

The abovementioned mechanism is also valid for F8. If a solvent slowly evaporates, the polymer backbones adopt the most stable, fully planar conformation [19–21]. Unlike P3ATs, F8 does not form a stacking structure because of the steric hindrance between adjacent alkyl chains. As a result, in β-phase thin films, ordered packing structures are not formed, and no clear X-ray diffraction peaks are observed [22, 32]. On the other hand, in thermally annealed thin films, crystallization of alkyl chains is preferred and thus the backbones adopt less stable, twisted conformations [33].

**Fig. 2:**
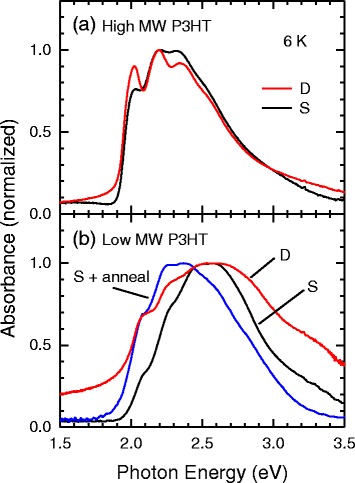
Normalized absorption spectra of thin films of **a** high and **b** low MW P3HTs at 6 K. *S* and *D* mean the samples prepared by spin-coating and drop-casting, respectively

Next, we show the absorption spectra of the prepared thin films of P3HT in Fig. 2. As shown in Fig. 2a, the absorption spectra of drop-cast and spin-coated thin films of high MW P3HT are the same as those in literature [34–37]. The absorption spectrum of spin-coated thin films of low MW P3HT is slightly blueshifted with respect to those of high MW P3HT. This blueshift is sometimes attributed to the shorter backbones but is largely reduced if the formation of form I is promoted by thermal annealing. This means that the true reason for the blueshift is the presence of a larger fraction of amorphous backbones in the samples [38]. On the other hand, the measured absorption spectrum of drop-cast thin films of low MW P3HT has a large baseline shift due to light scattering from the slightly rough surface. Unfortunately, from the spectrum, it is difficult to find the absorption bands specific to form II; this point will be discussed further later.

**Fig. 3:**
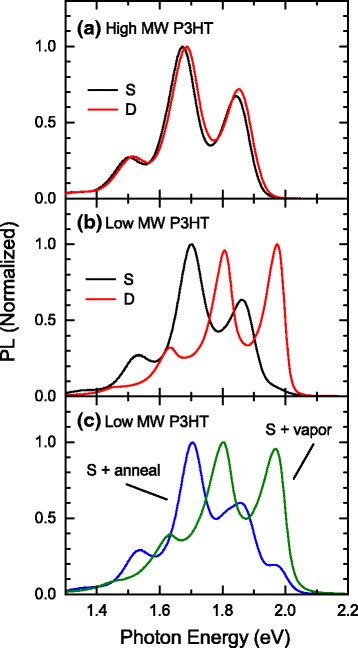
Normalized PL spectra of **a** high and **b**, **c** low MW P3HTs at 6 K. *S* and *D* mean the samples prepared by spin-coating and drop-casting, respectively

PL spectra of the prepared thin films are shown in Fig. 3. As shown in Fig. 3a, the PL spectral shape of thin films of high MW P3HT is also the same as those reported for form I of other polythiophene derivatives [34, 36, 39]. Interestingly, low MW P3HT exhibits the similar PL spectrum if the samples are prepared by spin-coating (see Fig. 3b). The observed PL can thus be attributed to form I and indicates that the spectral shape and the peak photon energy are virtually independent of the backbone length and are mainly determined by the packing structure. In contrast to those samples, drop-cast thin films of low MW P3HT show a PL spectrum that is blueshifted by more than 0.1 eV with respect to those of form I. Since the amorphous backbones of polythiophene derivatives exhibit much broader and featureless PL [35–37], the blueshifted PL is attributable to form II. In Fig. 3c, we also show the PL spectra of spin-coated thin films of low MW P3HT after thermal annealing or vapor treatment. In annealed (vapor-treated) samples, the PL component from form I (form II) is dominant but the other form is also present. Therefore, it can be concluded that for PL studies on the form II modifications, simply drop-cast samples are more suitable than the others.

The results in Fig. 3b give us valuable information regarding interchain interactions in π-conjugated polymers. Unlike small molecules [40, 41], it is not easy to obtain experimental evidence showing intermolecular (interchain) interactions in π-conjugated polymers. In the case of small molecules, intermolecular interactions can be investigated from simple comparisons between solid-state and solution samples. For example, the shift of the lowest excited state due to intermolecular interactions can be determined from the difference in the onset of PL. On the other hand, in π-conjugated polymers, the polymer backbones adopt different conformations in solid-state and solution samples, and the planarization of the polymer backbones also result in a redshift of PL [35–37]. As a result, the observed redshift of PL is not a direct evidence showing interchain interactions. In contrast, since the polymer backbones adopt fully planar conformations in form I and II modifications, comparisons between them allow us to focus on interchain interactions.

Since a π stack is distant from the adjacent one by more than 10 Å, the blueshift of PL in Fig. 3b is attributed to the increase in the stacking distance from 3.8 to 4.4 Å [2, 3, 6, 9]. In addition to the blueshift, the PL spectrum of form II has the slightly larger 0–0 transition at 1.98 eV. Spano and his group have developed a theoretical model, i.e., a weakly coupled H aggregate model, and have succeeded to explain several characteristic features of the PL of P3HT thin films (form I) such as the redshifted PL spectrum with respect to that of solution samples, the extremely low PL quantum efficiency, and the suppressed 0–0 transition [42–45]. In the past, these were believed to be caused by different factors. For instance, the redshift was attributed solely to the planarization of the backbone, the decrease in PL quantum efficiency was explained by efficient energy transfer into quench sites, and the suppressed 0–0 transition was ascribed to the reabsorption effect. Now, the weakly coupled H aggregate model has been widely accepted but there is still lack of clear experimental evidence of the model, i.e., interchain interactions. According to the model, in form II where interchain interactions are weakened due to the longer stacking distance, the slight blueshift of PL, the recovery of the 0–0 transition, and an increase in PL quantum efficiency with respect to those of form I are naturally expected. The former two expectations can be found in Fig. 3b, and the last one can be confirmed by the fact that the PL quantum efficiency of form II samples was triple that of form I in our measurements. Therefore, we believe that our comparison between the form I and II modifications could be an important evidence of interchain interactions in P3HT.

**Fig. 4:**
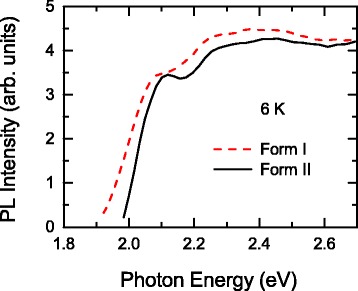
Excitation spectra of spin-coated and drop-cast thin films measured at 6 K

Finally, we show the excitation spectra of forms I and II at 6 K in Fig. 4. These excitation spectra were obtained by measuring PL intensities at 1.7 and 1.8 eV for form I and II, respectively. Although the excitation spectrum is not necessarily consistent with the absorption spectrum, in particular in the case of the sample consisting of several crystalline and amorphous components, the excitation spectra in Fig. 4 suggest that the absorption spectra of forms I and II are similar to each other. This spectral similarity is probably a reason why the characteristic absorption spectrum of form II cannot be seen in Fig. 2b.

The shift of the two excitation spectra is determined to be around 0.05 eV. This shift corresponds to half of the PL blueshift of 0.1 eV. The rest of the PL blueshift must be attributed to a decrease in the Stokes shift although the Stokes shift is not directly influenced by interchain interactions. However, the Stokes shift may depend on the strength of interchain interactions through the migration process of the excited states. Solid-state samples of π-conjugated polymers are not single crystals and can be regarded as ensembles of sites and crystalline domains with various energy levels. Thus, the excited states tend to migrate into sites and domains with lower energy levels prior to PL emission [46–48]. As a result, the observed Stokes shift depends on the distribution of the energy levels. In form I samples, the distribution of the energy levels is more greatly expanded by stronger interchain interactions compared to form II samples. It is thus reasonable to expect that the migration process within such the large energy level distribution results in the larger Stokes shift.

## Conclusions

In this work, we have prepared thin films of low and high MW P3HT using several fabrication techniques and compared their X-ray diffraction patterns and PL spectra. It has been found that simple drop-cast thin films of low MW P3HT exhibit the PL spectrum attributable to the form II modifications, having less inclusion of the other PL components. Since the polymer backbones adopt fully planar conformations in both form I and II modifications, the differences in PL properties between them can be attributed to the difference in the stacking distance. Therefore, the comparison between these PL spectra shows how interchain interactions influence the PL properties of P3HT in the solid state.

## Abbreviations

F8: Poly(9,9-dioctylfluorene)
MW: Molecular weight
P3AT: Poly(3-alkylthiophene)
P3BT: Poly(3-butylthiophene)
P3HT: Poly(3-hexylthiophene)
PDI: Polydispersity index
PL: Photoluminescence
XRD: X-ray diffraction
